# A novel nomogram for predicting cardiometabolic diseases from modifiable risks in middle-aged adults-implication for health education

**DOI:** 10.3389/fendo.2023.1291741

**Published:** 2024-01-24

**Authors:** Chia-Hao Chang, Ming-Shyan Lin, Yu-Chih Lin, Tung-Jung Huang, Mei-Yen Chen

**Affiliations:** ^1^ Department of Nursing, Chang Gung University of Science and Technology, Chiayi, Taiwan; ^2^ Department of Cardiology, Chang Gung Memorial Hospital, Chiayi, Taiwan; ^3^ Department of Family Medicine, Chang Gung Memorial Hospital, Yunlin, Taiwan; ^4^ Department of Pulmonary and Critical Care, Chang Gung Memorial Hospital, Yunlin, Taiwan; ^5^ School of Nursing, Chang Gung University, Taoyuan, Taiwan

**Keywords:** middle-aged, modifiable risks, health-promoting habits, cardiometabolic disease, nomogram, health education

## Abstract

**Background:**

Middle-aged adults often overlook critical modifiable risk factors that contribute to the emergence of cardiometabolic diseases (CMDs), including hypertension and diabetes. Many CMDs can be alleviated by addressing these modifiable risks. However, there has been insufficient research focused on rural adults with lower levels of health literacy in this regard. The aim of this study was to explore and develop an intuitive assessment tool for predicting cardiometabolic diseases (CMDs), which can be used for health education with adults of low health literacy.

**Methods:**

This was a community-based, cross-sectional study. A structured questionnaire on health-promoting habits, smoking, sleep, and physiological biomarkers was obtained via community health screening in the coastal region of Yunlin County, Taiwan. Multivariate logistic regression was used to screen for significant variables in the nomogram construction. Analysis with nonlinear restricted cubic spline was performed.

**Results:**

A total of 712 participants (60.9% females) aged 40–64 years, with middle school level or lower education, were included. The average age was 55.6 years (SD=7.3), and 220 individuals (31%) had CMDs. Multivariate logistic regression analysis revealed that age, lower scores of vegetables, fruit, water, and exercise (VFWE), smoking history, sleep deprivation, and being overweight were significantly associated with CMDs. The model incorporating these modifiable risk factors demonstrated good discriminatory ability, as indicated by an area under the receiver operating characteristic curve of 0.75 (0.73–0.76). A predictive nomogram was developed that presented modifiable risk factors in a simple graphical format to facilitate the prediction of CMDs.

**Conclusions:**

This study highlights a high prevalence of CMDs among middle-aged adults, along with the disregard for important risk factors that could be modified. The developed nomogram could be a practical and effective tool for community health education to enhance health literacy and prevent the progression of CMDs.

## Introduction

Middle-aged adults, typically aged between 40 and 64 years, are often called the “sandwich generation.” The term arises from the dual responsibility of caring for children and aging parents (1). As they navigate these roles, they face work demands and societal obligations. However, studies have indicated that healthy behaviors tend to decline in middle adulthood, increasing the risk of developing cardiometabolic diseases (CMDs), e.g., hypertension, diabetes, hyperlipidemia, and coronary heart disease (2, 3). For example, Sakib et al. (3) observed an increase in the prevalence of multimorbidity from 29.7% in the 45–49-year-old age group to 52% in individuals aged 60–64 years.

Empirical evidence has consistently demonstrated a strong association between CMDs, the occurrence of cerebrovascular disease (stroke), and cardiovascular events. CMDs are major contributors to global mortality, accounting for 32% of all deaths worldwide, with heart attack and stroke being the primary causes, representing 85% of CMD-related deaths. These acute events typically arise from blockages that impede blood flow to the heart or brain (4, 5). In Taiwan, CMDs account for a significant proportion of mortality and are the leading cause of cancer-related deaths. Stroke has consistently been ranked as the second leading cause of death in Taiwan for over three decades, imposing substantial burdens on affected families and society (6).

The existing literature highlights that most CMDs can be prevented by addressing lifestyle interventions and avoiding behavioral risk factors such as excessive alcohol consumption, tobacco use, unhealthy diet, physical inactivity, and obesity (7–9). A recent paradigm shift advocated by the American Heart Association, known as “life’s essential 8 (LE-8),” emphasizes the promotion of cardiovascular health (10, 11). LE-8 comprises five core health habits: maintaining a healthy diet, engaging in regular physical activity, avoiding nicotine exposure, and achieving an adequate body mass index and sleep health. It includes three ideal physiological indicators: levels of blood lipids, blood glucose, and blood pressure (4, 10). Literature indicates that lifestyle modifications, such as regular exercise, a healthy diet, smoking cessation, sufficient sleep quality, and weight management, can effectively prevent stroke, heart attacks, and the progression of CMDs and promote longevity (12–14).

Previous studies have established the pathways of CMDs and the dysregulation of systemic inflammation and metabolism, as well as their effects on atherosclerotic plaques and insulin resistance (15–17). Adopting primary prevention strategies can help mitigate accelerated inflammation associated with CMDs. These strategies include avoiding smoking, reducing alcohol consumption, engaging in physical activity, consuming adequate amounts of vegetables and fruits, ensuring sufficient water intake (>2000 mL/day), and maintaining a healthy weight (18–21). Although CMDs are a leading cause of death globally, research in this area has primarily focused on older adults, with limited studies examining and establishing easily understandable assessment tools for middle-aged individuals with CMDs, particularly those with lower health literacy. Therefore, this study aimed to investigate the association between modifiable risk factors and CMDs and to develop a nomogram format that provides an intuitive understanding of risk prediction for CMDs among rural adults.

## Materials and methods

### Design and population

In collaboration with a local hospital and health center, a community-based cross-sectional study was conducted between March and December 2022 in five townships located along the western coast of Yunlin County, Taiwan. Convenience sampling was used for participant selection. Before commencing the study, the institutional review board of the research ethics committee (IRB no: 202000109B0C102) and the approval of inform consent from each participant were obtained. The study selected community middle-aged adults who met the following criteria: (1) aged between 40 and 64 years and educational level ≦middle school (2), capable of communicating in either Mandarin or Taiwanese for one-on-one interviews, and (3) willing to sign the consent form. Participants who were unable to complete the questionnaires, those who independently walked to the community activity center, or individuals with incomplete laboratory data were excluded from the analysis.

### Measurement

*Demographics and cardiometabolic diseases* were assessed: (a) age, sex, education, marital status, living arrangement (alone, living with family, or living with friends), and health history of comorbidities diagnosed by a physician. Participants with hypertension, diabetes, hyperlipidemia, coronary heart disease, or stroke were classified as having CMDs; (b) height (cm) and body weight (kg) were measured while wearing light clothing and no shoes.

*Cardiometabolic risk factors* were assessed based on the previous reports (2, 10), including (a) control blood pressure (BP): the cutoff point of systolic/diastolic blood pressure (SBP/DBP) was >130/85 mmHg; (b) blood glucose control: We used glycated hemoglobin (HbA1C) ≥6% as the cutoff point of inadequate control level of blood glucose; (c) blood lipids control: serum triglyceride levels (TG) >150 (mg/dL) were classified as inadequate control.

*Modifiable risk factors* were assessed with a cluster of four health-promoting habits and three health-related behaviors that were based on the American Heart Association recommendations of LE-8 (2, 10) and previous studies (22–24).

(A)Health-promoting habits (including vegetables, fruits, water intake, and exercise, VFWE): Participants were asked to scale the frequency of healthy eating of vegetables, fruits, water intake, and practicing regular exercise on a four-point Likert scale, recorded as never (0), seldom (1), usually (2), and always (3). Do you (a) consume vegetables (V) ≥3 servings (1.5 bowls) per day; (b) consume fruit (F) 2 servings (1 bowl) per day; (c) consume water (W) ≥2000 mL per day; (d) practice regular exercise (E) ≥3 times, each for 30 min a week or 150 min per week with moderate sweating? The total score ranged from 0–12, with higher scores indicating better health-promoting habits.
(B)Ever-smoker: Participants were asked, “Do you smoke cigarettes or use e-cigarettes in recent months? with responses of “no: never” and “yes: former/current user.”
(C)Sleep deprivation: Based on the literature (25), participants were asked to answer this question: Currently or during the past week, have you experienced sleep distress or difficulty falling asleep? The responses were 0=never, 1=slightly, 2=ordinarily, 3=quite often, and 4=most often. Sleep deprivation was categorized as “no” (score 0–1) and “yes” (score 2–4).
(D)Healthy weight: Body mass index (BMI) was calculated using the standard formula (kg/m^2^), and BMI >24 was classified as overweight.

### Statistical analysis

**Figure 1:**
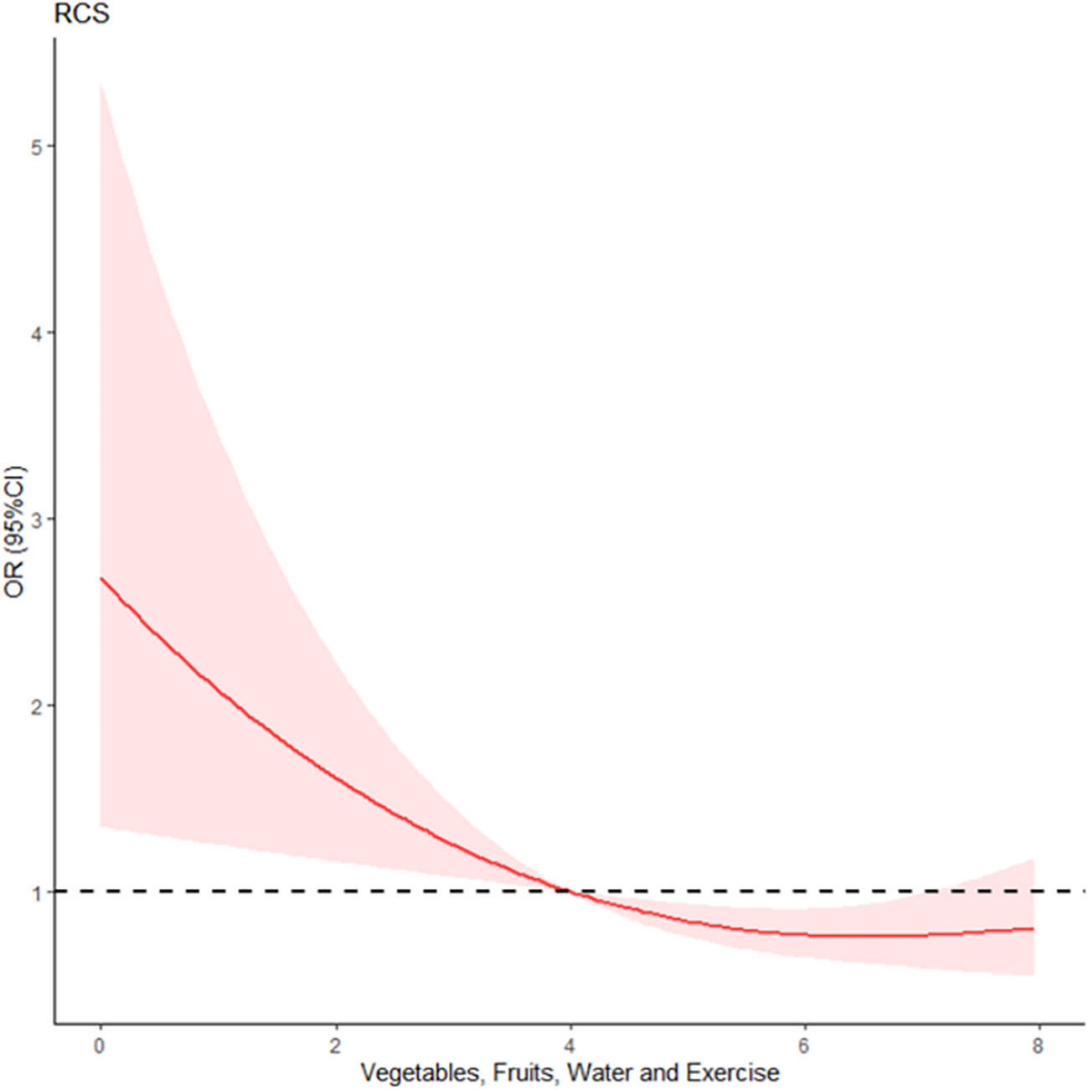
Non-linear correlation between VFWE (vegetables, fruits, water, and exercise) and cardiometabolic diseases. RCS, Restricted cubic spline of VFWE and CMDs.

Continuous variables were presented as mean (± SD); categorical variables were presented as percentages. Exceptions to blood pressure, blood glucose, and blood lipid levels were multicollinear with CMDs. We attempted to establish an understandable and predictive model between modifiable risk factors and age associated with CMDs. The correlations between CMDs and age, VFWE, smoking, sleep deprivation, and BMI were examined using a binary logistic regression model. Restricted cubic spline (RCS) analysis was performed to estimate the relationship between VFWE points and CMDs outcomes, adjusting for age, cigarette smoking, sleep deprivation, BMI, BP, HbA1C, and TG. We fitted a smooth continuous curve of adjusted odds ratio (OR) by RCS analysis with 95% confidence intervals (CI) across VFWE points, allowing for cubic form changes at the knot point. Thus, two linear relationships were generated (**Figure 1**). To test for the nonlinearity of the RCS curves, we checked the nonlinear p-value using Wald statistics. The p=0.03 implied that VFWE have a nonlinear relationship with the CMDs.

Furthermore, we used SiZer, an open-source R package that fits a degree 1 spline with one knot point using two-line piecewise-linear models. This package can check the critical (knot) point of the VFsWE, i.e., when the first derivative of a smooth continuous curve is significantly negative, possibly zero, or significantly positive across a range of smoothing bandwidths. The knot point is determined as follows:

$y_{i}=\beta_{0}+\beta_{1}x_{i}+e_{i}$ when $x_{i}\leq \text{α}$ and

$y_{i}=\beta_{0}+\beta_{1}x_{i}+\beta_{2}\left(x_{i}-\alpha \right)+e_{i}$ when $x_{i}>\text{α}$ where α is the knot point.

Because VFWE=3.95 has been reported as a knossst point, 4 was chosen for a categosrical split. Finally, to build an easy-to-use tool for predicting CMDs risk, each category of the five variables in the nomogram was given a point by representing ORs to a 0–100 “points” scale, and total points were calculated by summing these points. All statistical analyses were performed using R programming language (version 4.2.0) for Windows.

## Results

### Demographic characteristics and univariate analysis of modifiable risk factors associated with cardiometabolic diseases

A total of 712 participants aged 40–64 years who completed the community health screening were enrolled, of whom 434 (60.9%) were female and had attained education levels ≦middle school. Most of the participants (91%) lived with family or friends, 83.7% were married, the mean age was 55.6 (SD=7.3) years, and 220 (31%) participants were categorized as having CMDs. **Table 1** shows that among the participants with CMDs, 25% reported being ever smokers, 53% had sleep deprivation, 72% had BMI>24, 63% had SBP/DBP>130/85 (mmHg), 63% had HbA1C≥6%, and 41% had TG>150 (mg/dL). Regardless of age, adults with CMDs had significantly lower VFWE scores (p<0.05), were ever-smokers (p<0.05), had sleep deprivation (p<0.01), were overweight (p<0.01), and had abnormal blood pressure (p<0.01), HbA1C (p<0.01), and TG (p<0.01).

**Table 1:** Univariate analysis of demographic characteristics and potential modifiable factors associated with CMDs (N=712).

Variables	Cardiometabolic diseases (N %)			χ^2^/t	*p*
	No (n=492, 69%)		Yes (n=220, 31%)		
Age (years)	54.72±7.66		57.80±6.02	5.27	<0.01
VFWE (0–12)^1^	6.92±3.17		6.60±3.47	2.14	0.03
Ever-smoker				4.79	0.03
No	404 (82)		165 (75)		
Yes	88 (18)		55 (25)		
Sleep deprivation				14.25	<0.01
No	307 (62)		104 (47)		
Yes	185 (38)		116 (53)		
Body mass index (kg/m^2^)				21.14	<0.01
≦24	226 (46)		61 (28)		
24	265 (54)		159 (72)		
SBP/DBP (mmHg)^2^				13.74	<0.01
≦130/85	254 (52)		81 (37)		
130/85	236 (48)		139 (63)		
HbA1C (%)^3^				25.33	<0.01
<6	281 (57)		81 (37)		
6	210 (43)		139 (63)		
Triglyceride (mg/dL)				11.55	<0.01
≦150	354 (72)		130 (59)		
150	138 (28)		90 (41)		

^1^VFWE, vegetables, fruits, water, exercise; ^2^ SBP/DBP, systolic/diastolic blood pressure; ^3^ Glycated hemoglobin.

### Modifiable risk factors associated with CMDs

We observed a nonlinear association between VFWE and CMDs (p<0.05) in the RCS analysis (**Figure 1**), indicating a nonlinear relationship between VFWE and CMDs. We used SiZer, an open-ssource R package that fits a degree 1 spline with one knot point using two-line piecewise-linear models. The knot point (VFWE=3.95) with the maximum likelihood in the nonlinearity models in the RCS analysis was found. Therefore, a VFWE of four was chosen for the categorical split. We conducted a multivariate binary logistic regression analysis of modifiable risk factors associated with CMDs, and the results are presented in **Table 2**. In Model 1, all modifiable risk factors and age groups were included. Except for ever-smokers (p>0.05), the lower scores for health-promoting habits (VFWE, p<0.01), older age (p<0.001), sleep deprivation (p<0.001), BMI >24 (p<0.001), HbA1C ≥6% (p<0.01), and TG >150 (mg/dL) (p=0.05) were significantly associated with the log-odds of CMDs.

**Table 2:** Logistic regression of modifiable risk factors and age associated with cardiometabolic diseases.

Variables	Model 1			Model 2					
	Beta	SE	p	Beta	SE	p	OR	95% CI	Point
Age (years)	0.06	0.01	<0.001	0.06	0.15	<0.001	2.12	1.57-2.86	0-78
VFWE (4-0)^1^	-0.28	0.10	<0.01	-1.08	0.36	<0.01	0.34	0.17-0.69	44-100
Ever-smoker (1= yes)	0.30	0.21	0.16	0.43	0.21	0.04	1.53	1.02-2.32	47, 68
Sleep deprivation (1= yes)	0.59	0.18	<0.001	0.61	0.17	<0.001	1.84	1.31-2.60	47, 77
BMI (1 >24 kg/m^2^) ^2^	0.67	0.19	<0.001	0.86	0.18	<0.001	2.38	1.64-3.40	47, 89
SBP/DBP (1>130/85 mmHg)^3^	0.37	0.18	0.06						
HbA1C (1 ≥ 6 %)^4^	0.53	0.18	<0.01						
TG (1 > 150 mg/dL)^5^	0.37	0.19	0.05						

^1^VFWE, vegetables, fruits, water, exercise; ^2^BMI, body mass index; ^3^SBP/DBP, systolic/diastolic blood pressure; ^4^HbA1C, glycated hemoglobin; ^5^TG, triglyceride.

In Model 2, after adjustment with blood pressure, blood glucose, and blood lipids, the result showed that exception age, a lower score of VFWE (OR=0.34, 95% CI=0.17–0.69), ever-smoker (OR=1.53, 95% CI=1.02–2.32), sleep deprivation (OR=1.84, 95% CI=1.31–2.60) and overweight (OR=2.38, 95% CI=1.64–3.40) were significantly associated with CMDs.

### Construction of CMDs prediction nomogram

**Figure 2:**
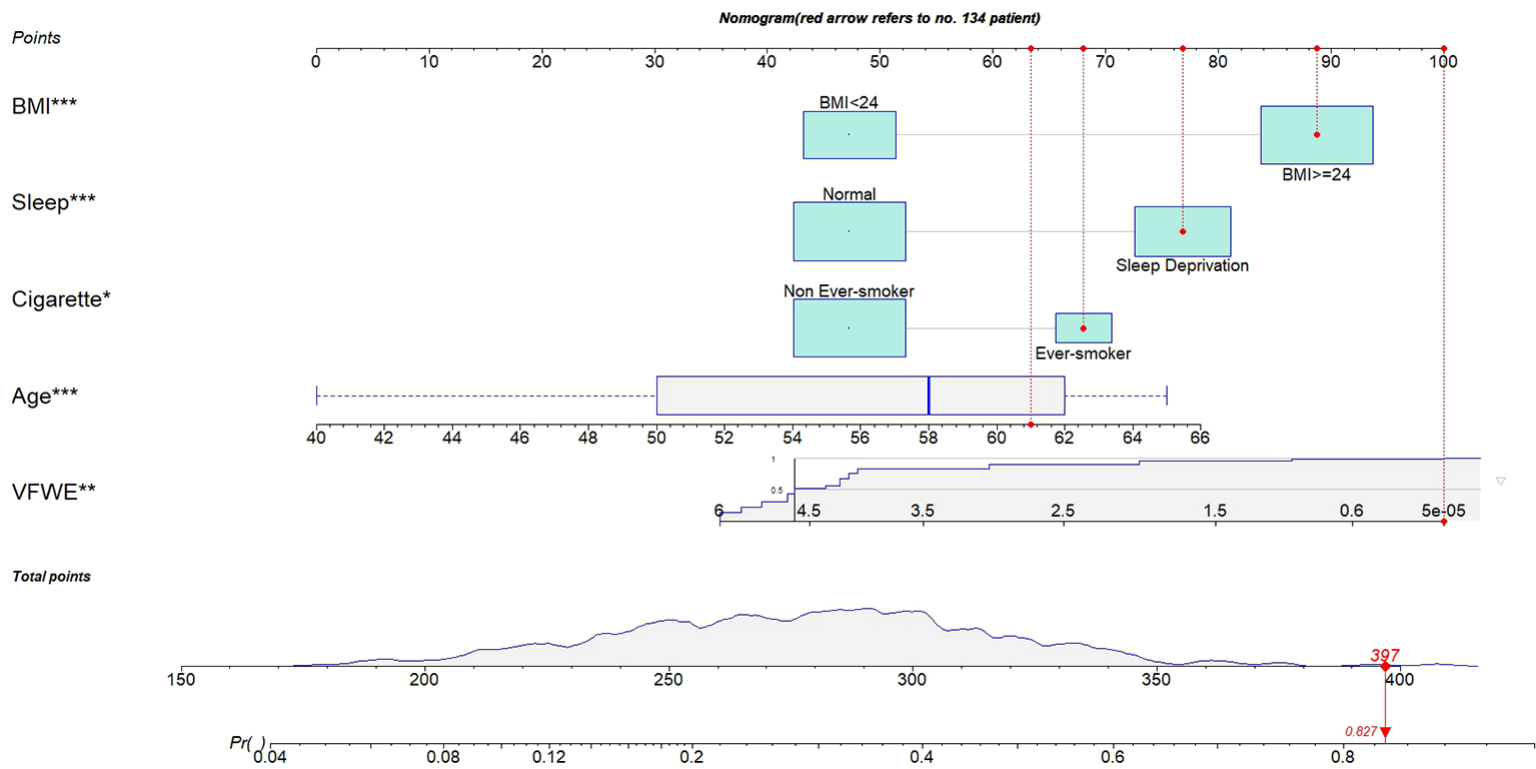
The nomogram was used to predict CMDs probability. A specific resident (case no 134) was shown to illustrate how to use the nomogram. This case of no 134 who had a BMI greater than 24, smoker, sleep deprivation, age of 61 and the score of VFWE was 0. The specific resident’s point corresponding to each covariate was presented at the top, and the total points were obtained from the sum of the points corresponding to each variable by a red dot. Once we obtained values from the 5 variables, the subject can be intuitively mapped onto the nomogram. From nomogram, we observed that the total points of case no 134 was 397, and the corresponding probability of CMDs was 0.827. BMI: body mass index; CMDs: cardiometabolic diseases; VFWE: vegetable, fruit, water, and exercise.

To predict modifiable risk factors for CMDs, we constructed a nomogram based on five variables (age, VFWE, ever-smoker status, sleep deprivation, and BMI) that were significant in **Model 2**. The points for each variable are listed in the right column of **Table 2**. By summing the points for the five variables, we derived the total number of points that changed from 185 to 412. The nomogram shows the modifiable risks of CMDs based on total points (**Figure 2**). For example, the risk of CMDs was lower than approximately 20% for those below 255 points and higher than approximately 65% for those with over 350 points. Additionally, if the logistic regression model predicts a probability of 0.827 for case number 134, it indicates an 82.7% likelihood that CMDs will occur based on the provided input variables (BMI greater than 24, smoking status, sleep deprivation, age of 61, and a VFWE score of 0).

### Risk stratification

**Figure 3:**
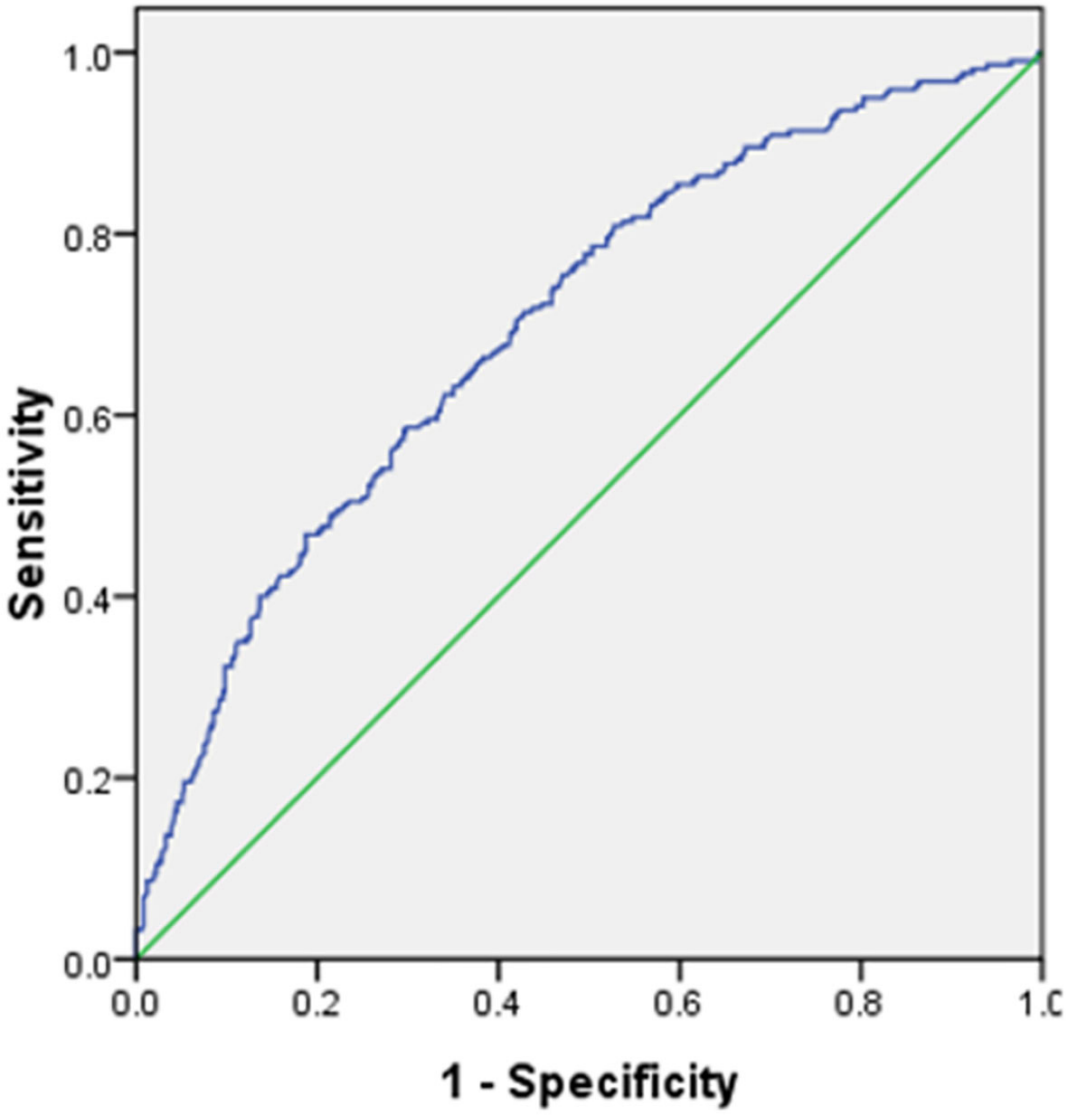
Assessing the discrimination of a fitted logistic model, via the ROC curve.

The discriminatory performance and cutoff probability of risk stratification are commonly quantified by measuring the area under the receiver operating characteristic curve (AUC). Overall, the discriminatory performance of the full model revealed an AUC of 0.75 (0.73–0.76, p<0.001) (**Figure 3**), indicating the suitability of this model in identifying participants with high risks of CMDs. Currently, there is a lack of research examining the efficacy of utilizing only health habits for the prevention and treatment of CMDs. Typically, the evaluation of CMDs relies on indirect functional and physiological assessments, historically presenting challenges attributed to technical limitations. Nevertheless, healthcare providers can proficiently leverage health habits to enhance cardiometabolic health literacy and to provide guidance for therapies aimed at the prevention and treatment of CMDs. The prevailing approach in investigating this association utilized BMI categories as the predictor variable. In their meta-analysis, Darbandi (26) integrated 38 cross-sectional and 2 cohort studies, encompassing a participant range of 105 to 137,256 individuals aged 18 or older. The collective AUC for BMI was 0.66 (95% CI, 0.63–0.69), which is comparatively lower than the AUC of 0.75 (95% CI, 0.73–0.76) observed in our study.

**Figure 4:**
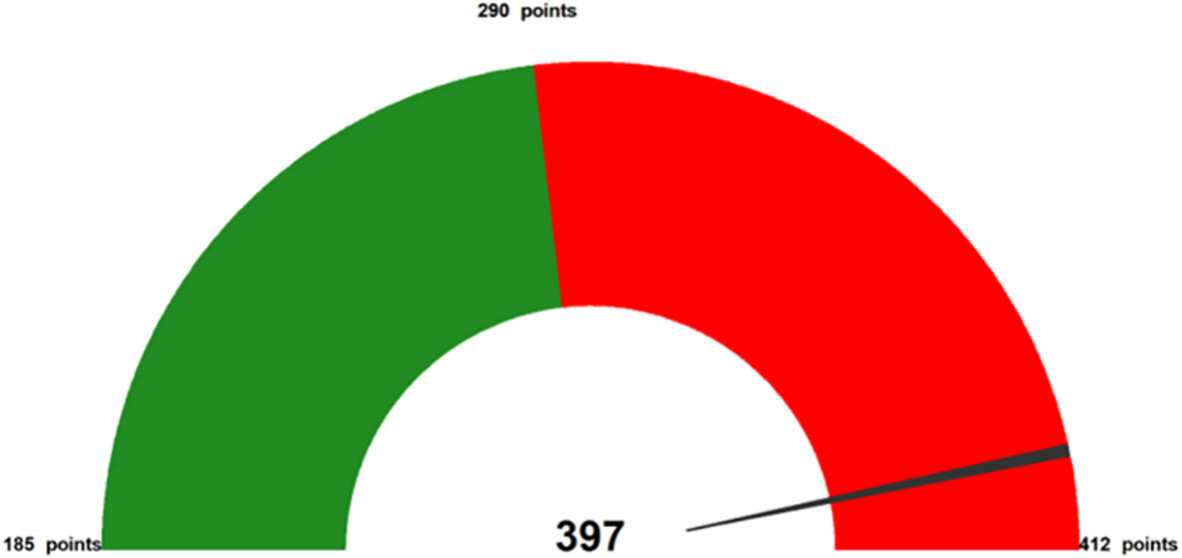
Example (case no. 134) as the representative of total points from nomogram. The above figure offers a specific case of how to represent a subject’s CMDs assessment with the five variables. The gauge at the top corresponds to the subject’s total point (which can range from 185–412 points, 290 points is optimal cutoff), with higher total point shown toward the right (a “full tank” of CMDs) in red. Thus, identifying VFWE and the other modifiable risks on which the subject can judge to near a full tank of CMDs. The total points of predicting the risk of CMDs are the sum of the 5 variables.

In order to determine the optimal cutoff point for the ROC curve, one commonly used method is the Youden index (27). The calculation formula for the Youden index involves finding the maximum value of sensitivity plus specificity minus 1. Consequently, this method aligns with the maximum sum of sensitivity and specificity. **Figure 4** provides a gauge of CMDs assessment, which illustrates the risk stratification based on the nomogram model by thresholding with the optimal cutoff predicted risk (the point-to-probability nomogram in **Figure 2** demonstrate that a probability value of 0.35 corresponds to a total of 290 points on the nomogram). The two risk intervals were defined as low- and high-risk groups for CMDs that were more understandable for health education. In our data, approximately 62.8% of the participants were classified into the low-risk group and 37.2% into the high-risk group.

## Discussion

This study yielded four important findings. First, nearly one-third of middle-aged rural adults reported having CMDs. Second, adults with CMDs have a high prevalence of modifiable risk factors, such as adopting few health-promoting habits, smoking, being overweight, sleep deprivation, and abnormal physiological biomarkers. Third, results suggest that significant reductions in CMDs can be achieved by encouraging individuals to start consuming vegetables and fruits, paying attention to hydration, and initiating physical activity without needing to reach a high frequency at which it becomes difficult to implement. Fourth, a novel nomogram with a simple graphical format for predicting the risk of CMDs was established, which can be used by primary healthcare providers to increase health literacy and prevent the progression of CMDs.

The present study showed that many participants reported knowing about the diagnosis of CMDs. However, many did not adopt health-promoting habits, such as eating adequate amounts of vegetables, fruits, drinking adequate amounts of water, and practicing regular exercise. Additionally, many of their cardiometabolic biomarkers remained at abnormal levels, such as body mass index, blood pressure, blood glucose, and blood lipids. Adults with known CMDs remain less likely to adopt health-promoting habits or LE 8 (2, 10). Possible reasons might be the low health literacy about cardiometabolic health and the limited quality of health resources provided by healthcare providers in rural areas. Further studies should consider enhancing cardiometabolic health literacy through innovative strategies and providing feasible continued education related to the new paradigm of cardiometabolic health for local healthcare providers.

Not surprisingly, these findings were consistent with those of previous studies. For example, in the United States, Lloyd-Jones et al. (10) pointed out that based on the LE8, the prevalence of ideal cardiovascular health is very low in all age groups (<1%) and 11% among the middle-aged group. In the United Kingdom, Stefan et al. (28) also stated that among the 20 leading global risk factors for years of life lost, high blood pressure, BMI, and blood glucose levels were the top risk factors for CMDs. Furthermore, previous studies have reported that most cardiometabolic events, such as myocardial infarction and stroke, can be prevented by adopting a healthy lifestyle and managing known risk factors (2, 8, 11). Hence, providing comprehensive, evidence-based behavioral counseling in primary care settings is a recommended first-line approach for promoting healthy behaviors and preventing worsening cardiovascular outcomes in adults with cardiovascular risks, especially in middle age (2). Thus, initiating a simple assessment tool to increase health literacy and prevent the progression of cardiometabolic diseases is an important strategy.

Promoting health literacy is a crucial approach within primary healthcare settings. Traditional educational manuals frequently play a significant role in the prevention and treatment of cardiometabolic diseases. Individuals with limited educational backgrounds may find understanding and identifying information in text to be abstract and challenging. As a result, healthcare providers, especially those dealing with demanding work schedules, carry a considerable workload. The present study’s strength is the application of a nomogram format to establish a simple, evidence-based model for predicting the risk of CMDs. We found that a novel nomogram with a simple graphical format to predict the risk of CMDs was easy to determine and offered each middle-aged adult to represent their CMD risk score with the sum of five variables. This research presents an innovative nomogram featuring a simple graphical format, aiming to enhance visual comprehension for individuals with limited health literacy or those living in rural areas. Furthermore, there is an expectation that this tool will substantially decrease the time needed for health education.

Healthcare providers can also combine the gauge reflecting each adult’s total points (ranging from 185 to 412 points). Further studies can apply this nomogram tool and gauge figures in an interesting board game or serious game to assess the total points for each middle-aged adult’s health-related lifestyle and quickly reflect the value of changing modifiable risks to prevent the progression of CMDs.

### Limitations

This study had some limitations. First, its cross-sectional design limits the causal relationship between modifiable risk factors and cardiometabolic diseases. Hence, a prospective design is necessary for future research. Second, we conducted this study in only one county and used nonrandom sampling, which might limit the generalizability of these findings. Third, health-promoting habits were based on self-report and recall bias, which may have resulted in inaccurate estimations. Fourth, the total points of the nomogram in this study ranged from 185 to 412, which is not a clinically convenient value. Simplification of these values will rely on a larger sample size in the future.

## Conclusions

Managing the progression of CMDs is difficult but important. A high prevalence of CMDs and many neglect-modifiable risk factors have been found among middle-aged rural adults. A novel nomogram with a simple graphical format was established to predict the risk of CMDs, which healthcare providers can easily use it to increase cardiometabolic health literacy.

## Data availability statement

The raw data supporting the conclusions of this article will be made available by the authors, without undue reservation.

## Ethics statement

The studies involving humans were approved by institutional review board of the Chang Gung Memorial Hospital Foundation (IRB no: 202000109B0C102). The studies were conducted in accordance with the local legislation and institutional requirements. The participants provided their written informed consent to participate in this study.

## Author contributions

C-HC: Methodology, Software, Writing – original draft. M-YC: Conceptualization, Methodology, Project administration, Writing – original draft. M-SL: Conceptualization, Data curation, Investigation, Writing – original draft. Y-CL: Data curation, Investigation, Writing – original draft. T-JH: Conceptualization, Investigation, Supervision, Writing – original draft.
